# Draft Genome Sequence of *Brevibacillus laterosporus* OSY-I_1_, a Strain That Produces Brevibacillin, Which Combats Drug-Resistant Gram-Positive Bacteria

**DOI:** 10.1128/genomeA.01093-17

**Published:** 2017-10-12

**Authors:** Xu Yang, En Huang, Mustafa Yesil, Lingzi Xiaoli, Edward G. Dudley, Ahmed E. Yousef

**Affiliations:** a Department of Food Science and Technology, The Ohio State University, Columbus, Ohio, USA; b Department of Environmental and Occupational Health, University of Arkansas for Medical Sciences, Little Rock, Arkansas, USA; c Department of Food Science, The Pennsylvania State University, University Park, Pennsylvania, USA; d Department of Microbiology, The Ohio State University, Columbus, Ohio, USA

## Abstract

*Brevibacillus laterosporus* OSY-I_1_ is a Gram-positive spore-forming bacterium isolated from soil. The bacterium produces brevibacillin, an antimicrobial lipopeptide effective against several drug-resistant Gram-positive bacteria. Here, we present the draft genome sequence of the strain OSY-I_1_ and the gene cluster responsible for the biosynthesis of brevibacillin.

## GENOME ANNOUNCEMENT

The need for new antimicrobial compounds that are effective against antibiotic-resistant pathogens is on the rise. According to the U.S. Centers for Disease Control and Prevention (CDC), more than two million people suffered from antibiotic-resistant bacteria with 23,000 annual deaths, and these estimates did not even include people who died from other diseases that were complicated by antibiotic-resistant bacterial infections (1). Brevibacillin, an antimicrobial lipopeptide effective against multidrug-resistant strains, was discovered in 2016, and its structure was fully elucidated (2). The compound was isolated from a soil microorganism, *Brevibacillus laterosporus* OSY-I_1_, which is a Gram-positive spore-forming bacterium. Based on a follow-up study (3), brevibacillin binds to lipoteichoic acid of the Gram-positive cell wall and further exerts its membrane disruption functions to cause cell leakage. To better understand the biosynthetic pathway of brevibacillin, the draft genome sequence of *B. laterosporus* OSY-I_1_ was determined.

The genomic DNA of OSY-I_1_ was prepared using the DNeasy blood and tissue kit (Qiagen, Valencia, CA). Concentration of extracted DNA was determined by NanoDrop spectrophotometer (ND-1000; Thermo, Fisher Scientific, MA). The Illumina Nextera v2 kit was used to construct the paired-end library for OSY-I_1_, followed by the Illumina Miseq next-generation sequencer (500-cycle) to generate 2 × 250 bp paired-end reads. The raw reads were later *de novo* assembled by SPAdes genome assembler (v3.10.1) (4), which generated 173 contigs, with a maximum contig size of 558,482 bp. The resulting draft genome, based on assembled contig information, revealed a genome size of 5,176,419 bp, and an average G+C content of 40.27%, calculated by Artemis software (5). Annotation of the draft genome was performed by the Rapid Annotations using Subsystems Technology (RAST) server (6). Annotation results revealed 4,823 coding sequences (CDS) in the draft genome, with putative functions assigned to 68% CDS. The OSY-I_1_ chromosome also contains 111 tRNA genes and one transfer-messenger RNA (tmRNA) gene, as predicted by software Aragon (7), and carries 2 vancomycin-resistant operon genes which may synthesize peptidoglycan with altered structure (8). However, OSY-I_1_ tested sensitive to vancomycin (MIC 1 μg/ml), indicating both antibiotic-resistant operons may be dysfunctional (9).

The average nucleotide identity (ANI) values between OSY-I_1_ and four sequenced *B. laterosporus* genomes were calculated using JSpecies software (10). Results indicated that OSY-I_1_ shared highest (89.34%) similarity with *B. laterosporus* B9, followed by *B. laterosporus* PE36 (89%), *B. laterosporus* LMG 15441 (88.96%), and *B. laterosporus* GI-9 (88%).

The gene cluster responsible for brevibacillin biosynthesis was identified from the draft genome sequence of OSY-I_1_. Most of the biosynthetic gene cluster was encoded on three contigs, and gaps were filled by PCR and Sanger sequencing. The complete brevibacillin gene cluster consisted of five nonribosomal peptide synthetases and an ABC transporter. In addition, a complete tyrocidine A gene cluster was discovered, indicating that OSY-I_1_ is capable of producing multiple antimicrobial compounds.

### Accession number(s).

The draft genome sequences of *Brevibacillus laterosporus* OSY-I_1_ and the brevibacillin biosynthetic gene cluster have been deposited at the NCBI GenBank database under accession numbers NOLX00000000 and MF526970, respectively. The versions described in this paper are NOLX01000000 and MF526970.1, respectively.
